# Intravenous ferric carboxymaltose versus oral ferrous sulphate for the treatment of moderate to severe postpartum anaemia in Nigerian women (IVON-PP): protocol for an open-label randomised controlled type 1 hybrid effectiveness-implementation trial

**DOI:** 10.1136/bmjopen-2024-086553

**Published:** 2024-08-14

**Authors:** Bosede Bukola Afolabi, Victoria Olawunmi Adaramoye, Titilope Adenike Adeyemo, Mobolanle Balogun, Eleanor J Mitchell, Kate Walker, Opeyemi Rebecca Akinajo, Ibraheem Ajibola Abioye, Aduragbemi Banke-Thomas, Ochuwa Adiketu Babah, Chisom Florence Chieme, Yewande Oshodi, Rachel Quao, Ejemai Amaize Eboreime, Folasade Ogunsola

**Affiliations:** 1 Department of Obstetrics and Gynaecology, University of Lagos College of Medicine, Lagos, Nigeria; 2 Department of Obstetrics and Gynaecology, Lagos University Teaching Hospital, Surulere, Nigeria; 3 Department of Hematology & Blood Transfusion, University of Lagos College of Medicine, Lagos, Nigeria; 4 Department of Community Health & Primary Care, University of Lagos, Mushin, Nigeria; 5 Nottingham Clinical Trials Unit, University of Nottingham Faculty of Medicine and Health Sciences, Nottingham, UK; 6 University of Nottingham, Nottingham, UK; 7 Lagos University Teaching Hospital, Surulere, Nigeria; 8 Department of Global Health and Population, Harvard University T H Chan School of Public Health, Boston, Massachusetts, USA; 9 Maternal Adolescent Reproductive and Child Health Centre, London School of Hygiene & Tropical Medicine Faculty of Infectious and Tropical Diseases, London, UK; 10 Centre for Clinical Trials, Research, and Implementation Science, College of Medicine, University of Lagos/ Lagos University Teaching Hospital, Idi-araba, Lagos, Nigeria; 11 Department of Psychiatry, University of Lagos College of Medicine, Lagos, Nigeria; 12 Department of Planning, Research & Statistics, NPHCDA, Abuja, Nigeria; 13 Department of Psychiatry, University of Alberta Faculty of Medicine and Dentistry, Edmonton, Alberta, Canada; 14 Department of Microbiology, College of Medicine, University of Lagos/Lagos University Teaching Hospital, Akoka, Nigeria

**Keywords:** quality of life, depression & mood disorders, postpartum women, anaemia

## Abstract

**Introduction:**

Postpartum anaemia is often caused by iron deficiency with onset during the antepartum period and can be exacerbated by excessive blood loss at birth. Its prevalence is estimated as 50–80% in low-income and middle-income countries. It poses adverse consequences on the mother and negatively impacts her ability to care for her newborn. Prompt treatment of postpartum anaemia is thus important. Adherence to oral iron is reportedly low in Nigeria due to its side effects and forgetfulness by the mothers. Intravenous iron such as ferric carboxymaltose, given as a single dose, might help overcome adherence issues, but investigation in a high-quality randomised control trial in Nigeria is first required while evaluation of challenges around its implementation is also warranted.

**Objective:**

To determine the clinical effectiveness, tolerability and safety, of using intravenous ferric carboxymaltose (intervention) vs oral ferrous sulphate (control) for treating moderate to severe iron deficiency anaemia in postpartum women and to evaluate implementation of ferric carboxymaltose in treating postpartum anaemia in Nigeria.

**Methods and analysis:**

This study is an open-label randomised controlled trial with a concurrent implementation study. It is a hybrid type 1 effectiveness-implementation design conducted in four states across Northern and Southern Nigeria. A total of 1400 eligible and consenting women with postpartum moderate to severe anaemia (haemoglobin concentration <100 g/L) will be randomised to intravenous ferric carboxymaltose; a single dose at 20 mg/kg to a maximum of 1000 mg infusion administered at enrolment (intervention) or oral ferrous sulphate; 200 mg (65 mg elemental iron) two times per day from enrolment until 6 weeks postpartum (control). The primary outcome, proportion of participants who are anaemic (Hb <110 g/L) at 6 weeks postpartum will be analysed by intention-to-treat. Haemoglobin concentration, full blood count, serum iron, serum ferritin, transferrin saturation and total iron binding capacity will be measured at specific intervals. Implementation outcomes such as acceptability and feasibility of using ferric carboxymaltose for postpartum anaemia treatment in Nigeria will be assessed.

**Ethics and dissemination:**

This study is approved by the ethics committee of the teaching hospitals, Ministry of Health of the four states as required, National Health Research Ethics Committee and the drug regulatory agency, National Agency for Food and Drug Administration and Control (NAFDAC). Findings of this research will be presented at conferences and will be published in international peer-reviewed journals and shared with stakeholders within and outside Nigeria.

**Trial registration number:**

International standard randomised controlled trial number: ISRCTN51426226.

**STRENGTHS AND LIMITATIONS OF THIS STUDY**

- Multicentre randomised controlled trial conducted across diverse regions of Nigeria.
- Hybrid design evaluating effectiveness as well as implementation contexts and outcomes.
- Open-label design.
- Self-reported measures of adherence and some secondary outcomes.
- Generalisability is limited to similar populations and health systems.

## Introduction

### Background and rationale

 Maternal anaemia is a major public health burden with a high incidence in low-income and middle-income countries (LMICs).^1 2^ Iron deficiency causes more than half of all cases.^1^ Postnatal anaemia may result from untreated antenatal anaemia or blood loss around the time of birth.^3^ Its prevalence in LMICs has been estimated to range from 50% to 80%.^4^ A study in Eastern Nigeria found anaemia at 48 hours and 6 weeks postpartum to be 73% and 48%, respectively, in a cohort of 202 women followed from late pregnancy to 6 weeks postpartum.^5^ The consequences of postnatal anaemia are substantial. Postnatal anaemia increases the risk of infection and poor wound healing, and may cause fatigue and/or depression in the mother.^6^ It also adversely affects breastfeeding due to insufficient milk production and can reduce bonding between women and their newborn because of weakness and fatigue.^3 4 7^

Oral iron is used routinely for the treatment of mild to moderate anaemia soon after birth and up to 42 days, while blood transfusion is offered for severe anaemia or symptomatic women with moderate anaemia.^3^ Oral iron, though inexpensive, causes significant gastrointestinal adverse effects such as vomiting, constipation, diarrhoea and abdominal pain.^8^ This limits adherence to iron supplementation and poses a challenge to achieving optimal and timely correction of anaemia, as has been shown among pregnant women.^9 10^ In a large population-based study in 22 Sub-Saharan African countries, only 22.9% of Nigerian women had greater than 90 days of iron supplementation during pregnancy with their most recent birth.^11^

Whether intravenous iron can be a suitable alternative to oral iron for the treatment of postnatal anaemia is unclear. Intravenous iron can be given as a single dose and is suitable for patients who respond poorly to oral iron and are unable to tolerate the side effects, and moderately anaemic women that require more rapid iron replacement.^3^ Intravenous iron corrects anaemia faster and in fewer doses than oral iron and requires fewer patient-provider interactions.^1^ Intravenous iron is therefore optimal for LMICs where women do not routinely attend postnatal follow-up visits.^1^ Previous randomised trials of intravenous iron for postpartum anaemia have reported faster and more effective correction of anaemia compared with oral iron.^12^ These studies have been small, and none were conducted in an African setting.^12 13^ These studies also considered different formulations for intravenous iron including ferric carboxymaltose (FCM), iron sucrose and ferrous sulphate. ^13^FCM is convenient to administer as it can be administered in a single dose and is also associated with low amounts of potentially harmful non-transferrin bound iron.^13^ Thus, the safety and efficacy profile of FCM makes it an ideal formulation for this purpose.

This study aims to determine the effectiveness, tolerability and safety of intravenous FCM (intervention) vs oral ferrous sulphate (control) for treating moderate to severe iron deficiency anaemia in postpartum women (population). We also aim to evaluate implementation of FCM in treating postpartum anaemia in Nigeria. Our objectives are:

1. To determine the effectiveness of FCM vs oral ferrous sulphate in postpartum women with moderate to severe iron deficiency anaemia by conducting a randomised trial.
2. To determine the incidence of adverse drug events including the incidence of hypophosphataemia in the postpartum women and adherence to oral ferrous sulphate for treatment of postpartum anaemia.
3. To evaluate the acceptability and feasibility of using intravenous FCM in treating postpartum anaemia in Nigeria by conducting a concurrent implementation study.

## Methods and analysis

### Study design

**Figure 1:**
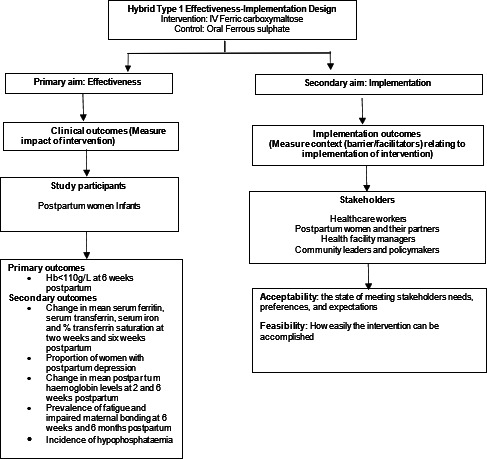
Flow chart of IVON-PP implementation-effectiveness hybrid type 1 trial design.

The IVON-PP trial uses a hybrid effectiveness-implementation design to assess intervention effectiveness and collect contextual information for implementation.^14^ A multicentre, parallel, open-label, superiority randomised controlled trial aimed to compare the effectiveness and safety of FCM (intervention) and ferrous sulphate (standard care) in treating iron deficiency anaemia among postpartum Nigerian women is being conducted. As the effectiveness of FCM is being assessed through various outcome measures, an implementation study will be conducted simultaneously to explore its acceptability and feasibility (implementation outcomes) (figure 1).

### Study sites

**Figure 2:**
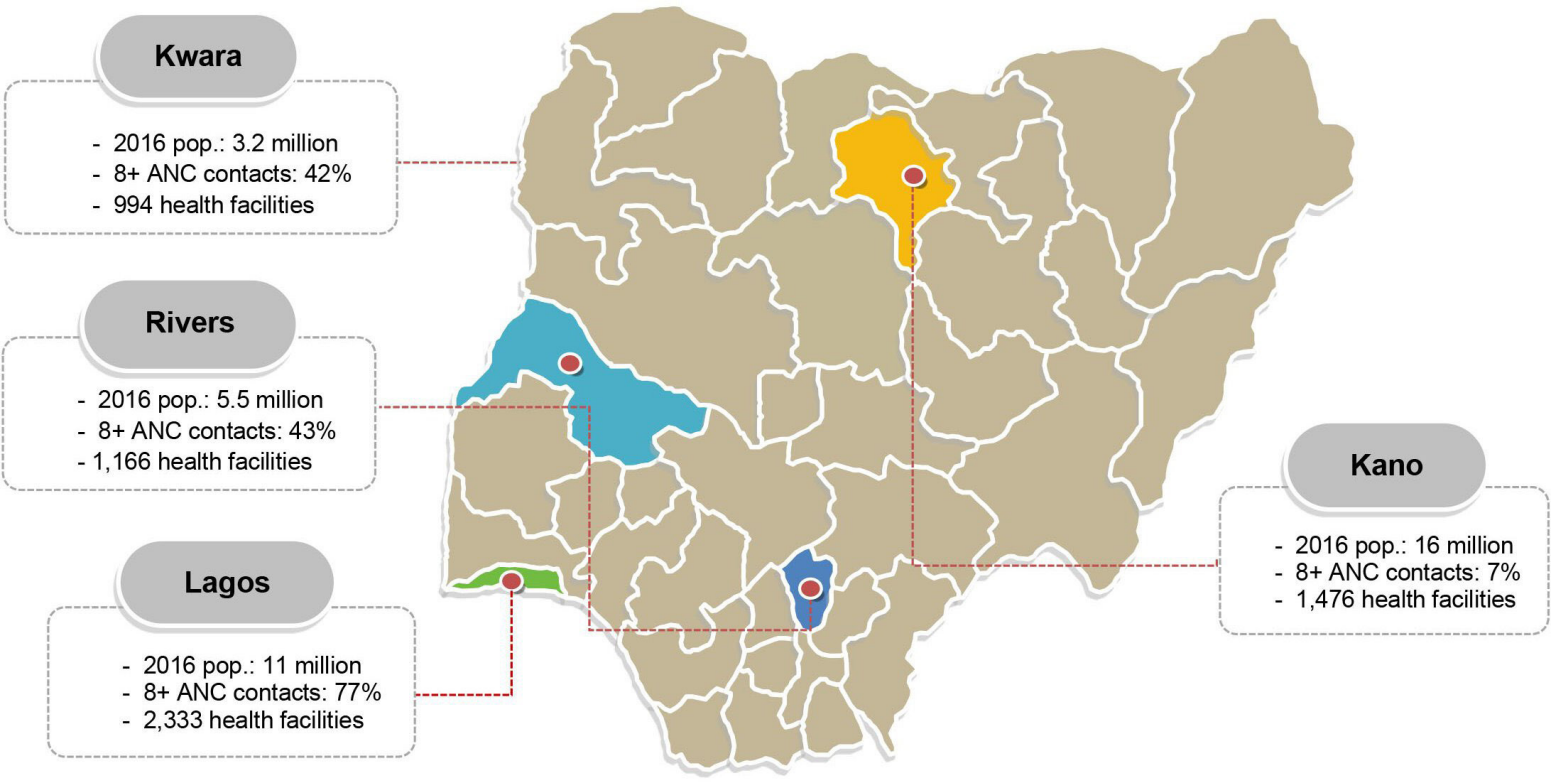
Map of Nigeria indicating the four selected study states with key population and service indices.

The study is being conducted in 20 recruiting sites in four states in Nigeria: Kano and Kwara states in the North, and Lagos and Rivers states in the South (figure 2). In each state, one tertiary, three secondary and one primary healthcare facilities are participating. The list of study sites can be obtained from the trial registry (ISRCTN51426226).

### Eligibility criteria

Inclusion criteria:

1. Women aged between 15 and 49 years and between 6 and 48 hours after birth.
2. Moderate or severe anaemia (haemoglobin <100 g/L), confirmed by Hemocue haemoglobinometer.
3. Able and willing to give written informed consent.

Exclusion criteria:

1. Blood transfusion, for any indication, within the last 3 months.
2. Symptomatic anaemia and a need for urgent correction.
3. Known haemoglobinopathy such as sickle cell disease, haemoglobin C disease.
4. Clinically confirmed malabsorption syndrome.
5. Known hypersensitivity or contraindication to any form of iron treatment, study drug or any of its excipients.
6. Self-reported pre-existing maternal depression or other psychiatric illness as evidenced by a YES response to any history of psychiatry ward hospitalisation, psychiatry medications, behavioural changes or past consultation with psychiatric services.
7. Severe allergic conditions such as severe asthma, eczema, or other atopic condition
8. Known autoimmune conditions for example, systemic lupus erythematosus, rheumatoid arthritis or known severe drug allergies.
9. Planning to move or reside outside the research area within the study period.

### Interventions

The intervention, FCM is given as a single dose of 20 mg/kg up to a maximum of 1000 mg diluted in 200 mL normal saline and infused over a minimum of 15–20 min. Eligible women randomised to the intervention arm (FCM) are administered the intravenous iron within 6–48 hours after delivery at the dedicated ward or daycare room, where she is also observed post infusion. The control drug, oral ferrous sulphate is given as one 200 mg tablet, which contains 65 mg of elemental iron, two times daily to be taken 1 hour before meals or 2 hours after meals with a full glass of water until 6 weeks postpartum. Women in both groups will have folic acid (5 mg daily) and vitamin C (200 mg two times per day). If a woman suffers severe adverse drug event up to grade 3, the study drug may be discontinued.

### Adherence

Women randomised to the control group are sent automated daily text messages as reminders to take their medication. They are asked to bring their pill sachets to each clinic or home visit for pill counting to calculate adherence. The empty pill packs are collected and labelled with the participant’s ID and archived at the trial site.

### Adverse drug event monitoring

Adverse drug events are closely monitored and recorded. At each clinic or home visit, women are asked of any symptom that has occurred and asked to report symptoms between study visits. Adverse drug events are documented according to the severity, duration, grade, relationship to trial drugs, interventions given and the outcome. The costs of treating all adverse drug events related to trial drugs are borne by the trial.

### Clinical outcomes

The primary outcome is the proportion of participants who are anaemic (haemoglobin < 110 g/L) at 6 weeks postpartum.

Secondary outcomes:

1. Change in mean serum ferritin, serum transferrin, serum iron and % transferrin saturation at 2 weeks and 6 weeks postpartum.
2. Proportion of women with postpartum depression, measured using the Edinburgh Postnatal Depression Scale (EPDS)^15^ at 6 weeks and 6 months postpartum.
3. Change in mean postpartum haemoglobin levels at 2 weeks and 6 weeks postpartum.
4. Achievement of a non-anaemic state (Hb ≥110 g/L) at 6 months postpartum.
5. Prevalence of moderate/severe anaemia at 6 weeks and 6 months postpartum. Moderate anaemia is defined as haemoglobin level 70–<100 g/L and severe anaemia as haemoglobin level <70 g/L.
6. Need for blood transfusion after iron treatment during the first 6 weeks postpartum.
7. Prevalence of fatigue at 6 weeks and 6 months postpartum, measured using the Fatigue Severity Scale (revised FSS-5R version).^16^
8. Proportion of women with secondary postpartum haemorrhage after treatment. This will be defined as excessive bleeding requiring surgical intervention or blood transfusion from 24 hours after delivery until 12 weeks postpartum.
9. Proportion of infants being breastfed (exclusive and any) at 6 weeks and 6 months postpartum.
10. Prevalence of impaired maternal-infant bonding at 6 weeks and 6 months postpartum measured using the Mother-to-Infant Bonding Scale.^17^
11. Incidence of confirmed or suspected maternal infection within 6 weeks of birth, as defined by a new prescription of antibiotics for presumed perineal wound-related infection, endometritis or uterine infection, urinary tract infection or other systemic infection (clinical sepsis).
12. Incidence of hypophosphataemia at 2 weeks and 6 weeks postpartum. We will measure vitamin D, alkaline phosphatase, procollagen type I N-terminal propeptide (P1NP), fibroblast growth factor 23 (FGF23), calcium, phosphate, all of which are biomarkers of phosphorus homeostasis and bone turnover. Hypophosphataemia is defined as serum phosphate level <2.5 mg/dL (0.81 mmol/L). Mild hypophosphataemia as 2–2.5 mg/dL (0.65–0.81 mmol/L), moderate as 1–2 mg/dL (0.32–0.65 mmol/L) and severe as <1 mg/dL (0.32 mmol/L).
13. Incidence of early neonatal death, defined as death of newborn from enrolment of the mother to before seven completed days.
14. Incidence of late neonatal death, defined as death of the newborn after seven completed days of birth to before 28 completed days.
15. Incidence of infant death, defined as death from enrolment before the age of 6 months.
16. Incidence of postnatal maternal death from enrolment up to 6 weeks and at 6 months postpartum.
17. Incidence of adverse drug events.
18. Quality of life measured using the WHO Quality of Life BREF^18^ at 6 weeks and 6 months postpartum.

### Implementation outcomes

1. Acceptability of the intervention to women and healthcare professionals.
2. Feasibility of implementing the intervention.

### Sample size

We used data from a recent meta-analysis,^19^ which reported that 39% of women were anaemic at 6 weeks postpartum, as the control event rate. A sample size of 697 participants per group would provide 90% power to detect a relative reduction of 20% for intravenous iron to reduce the event rate of anaemia at 6 weeks postpartum to 30%, after accounting for 10% loss to follow-up. Power calculations assume a 1:1 randomisation ratio to each of the two treatment arms. 1400 women will be enrolled into the trial.

### Recruitment

Randomisation and enrolment will occur between 6 and 48 hours after birth. Postpartum women within this timeframe (6–48 hours postpartum) are being screened for eligibility, provided with information about the trial, and given time to consider their participation. They are randomised to one of the two treatment groups after obtaining written informed consent.

### Randomization and allocation

Individual randomisation and allocation concealment are done using ‘Sealed envelope’ a web-based randomisation software; https://www.sealedenvelope.com/access/ in a 1:1 ratio in blocks stratified by study site.

### Blinding

It will be difficult to blind the treatment allocation as the intervention is administered intravenously, while the control is administered orally. However, the primary outcome is an objective measure determined by laboratory evaluation. For the secondary outcomes that are subjective: the EPDS, Quality of Life questionnaire, fatigue scale, Maternal Infant Bonding questionnaire and adverse event report form, the research nurses performing data collection for these outcomes will be blinded to group allocation. Data on treatment compliance will be collected only after all other outcome data for the woman’s visit has been completed and submitted.

Only the senior data manager has unblinded and full access to the database. The trial statistician has access to blinded data to support the Data Safety and Monitoring Board (DSMB) and Trial Steering Committee (TSC). All other investigators only have access to blinded summary reports.

### Participants’ timeline

**Figure 3:**
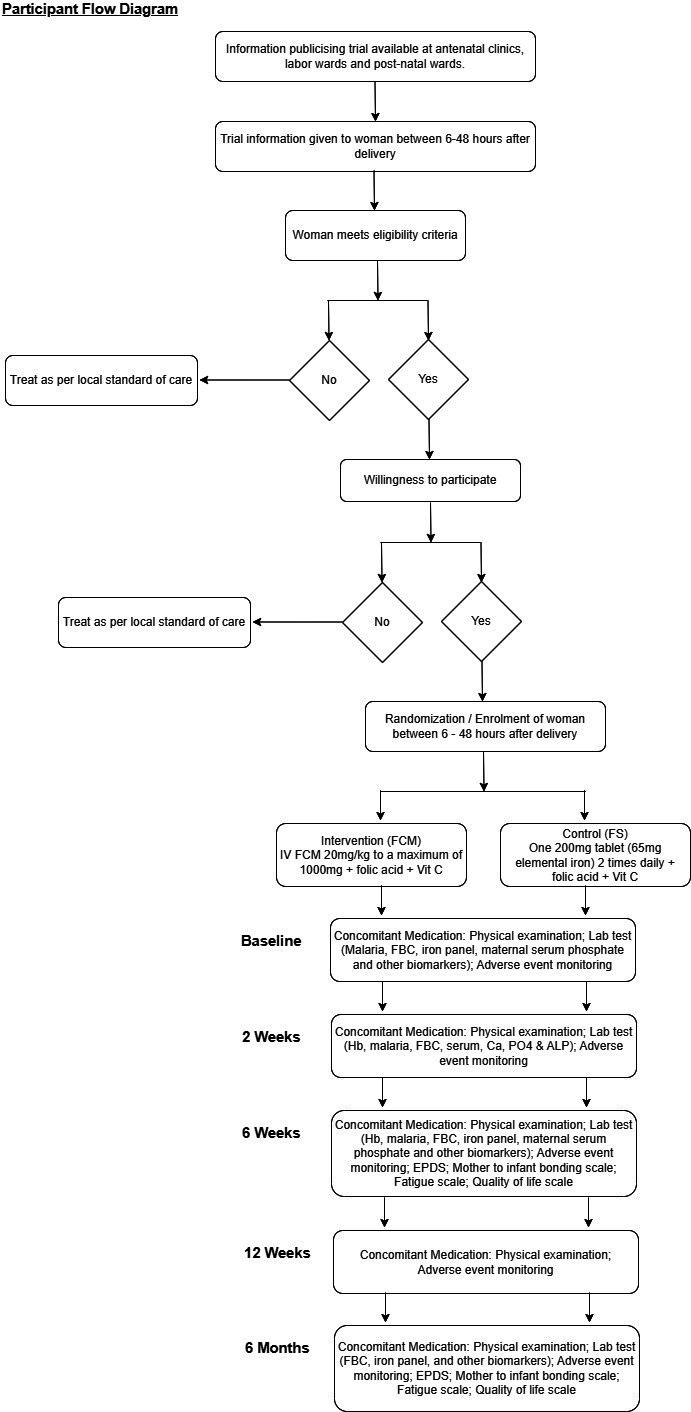
Participant flow diagram.

Women will be enrolled between 6 and 48 hours after birth and followed up until 6 months postpartum (figure 3).

### Retention strategies

To minimise loss to follow-up, counselling sessions are extended to partners and family members when necessary during follow-up visits. Reminders for follow-up appointments are sent via short message service and telephone. The details of participants’ home addresses are also collected at enrolment and reviewed before each visit. For participants who cannot be reached, community tracking is done (home-based monitoring system designed to locate participants in their homes or place of work). Consent for community tracking is obtained at enrolment.

### Data collection methods

REDCap (Research Electronic Data Capture V.13.8.3), developed by Vanderbilt University in Nashville, Tennessee, USA, is a secure web-based application used to capture data for clinical research, create databases and manage projects. It is being used to collect all relevant study data. Electronic case report forms, surveys and other data collection instruments were developed and refined in the study database to collect information at enrolment, follow-up, end of study visits and for adverse events. Other study instruments in the trial database are the EPDS, fatigue scale, MIB scale and quality of life scale e-forms. Designated investigators and study staff will enter the information in real time as required by the protocol into the electronic forms on the REDCap and source documents as indicated.

### Data management

Data are collected using electronic case report forms at various participant study visits using assigned participant IDs and uploaded in real-time to the REDCap database after being checked by the study site coordinator. Data management is guided by standard operating procedures that will be periodically revised and communicated with the study staff.

### Statistical methods and analysis

Primary analyses will be conducted with the intention to treat principle. Categorical variables will be expressed as frequencies and percentages. Continuous variables will be presented as means (SD) or medians (interquartile ranges) depending on whether they are normally distributed or not. The effect of intravenous iron (vs oral iron) on the primary endpoint will be analysed using log-binomial regression models to obtain relative risks, 95% CIs and p values. The impact of clustering by site will be evaluated by comparing to logistic generalised linear mixed models using likelihood ratio tests, and the more parsimonious model presented. The extent to which inclusion of baseline covariates modifies the precision of estimates will be explored. Generalised linear mixed models will be used to estimate the effect of the treatment arm on repeated measures of haemoglobin and iron status biomarkers. The proportion of participants who are anaemic or iron deficient, and the proportion of participants who achieve anaemia correction at 6 weeks and 6 months follow-up will be compared. Subgroup analyses will be conducted to compare study findings by region (Lagos, Kwara, Kano and Rivers), mode of birth (caesarean vs vaginal birth) and by the occurrence of primary postpartum haemorrhage. A two-tailed test of hypothesis will be performed based on an alpha level of 0.05. R and RStudio (2020)http://www.rstudio.com/ will be used for statistical analysis.^20^ An interim analysis will be conducted by an independent statistician after achieving 50% of the sample size and data presented to the Data Safety and Monitoring Board (DSMB). Based on O’Brien-Fleming method,^21^ the threshold for significance of the primary endpoint at the interim analysis will be alpha=0.0054. The clinical trial will be terminated if the null hypothesis is rejected. The alpha level for the analysis at the end of the study will be adjusted to 0.0492 based on alpha adjustment based on O’Brien-Fleming method.

### Data monitoring

The day-to-day management of the trial is the responsibility of the Trial Management Group (TMG) who ensure high-quality delivery of the trial in accordance with agreed timelines, including early identification of potential problems and mitigations. The TMG reports to the independent TSC who are responsible for oversight of the trial. The TMG includes the chief investigator, co-investigators, the trial manager and data manager. The independent DSMB meets at regular intervals throughout the trial; they aim to protect the trial’s validity and credibility. The DSMB are responsible for monitoring the trial data and they report to the TSC. The committee members met before the commencement of the study and they are to meet at least annually to review unblinded data, monitor the progress of the study, recruitment rate and to assess whether there are any significant safety concerns or ethical issues. If they find any, they make recommendations to the TSC regarding whether the trial should be halted, modified or continued as planned. The TSC reviews them carefully and consider the recommendations alongside other aspects of the trial’s progress. Based on this review, the TSC may decide to implement the recommendations, modify the trial protocols or take other appropriate actions to address any identified concerns. They monitor the progress of the trial by holding virtual meetings two times a year or as the need arises.

The clinical monitors conduct routine monitoring online and onsite every 2 weeks to ensure reported trial data are accurate, complete and verifiable from source documents, and that the conduct of the trial follows the currently approved protocol/amendment(s), with Good Clinical Practice, and applicable regulatory requirement(s).

Auditing of research records will be done by the monitoring committees as outlined above, while financial auditing will be done by the Research Management Office and Audit Department of University of Lagos.

### Implementation study

**Figure 4:**
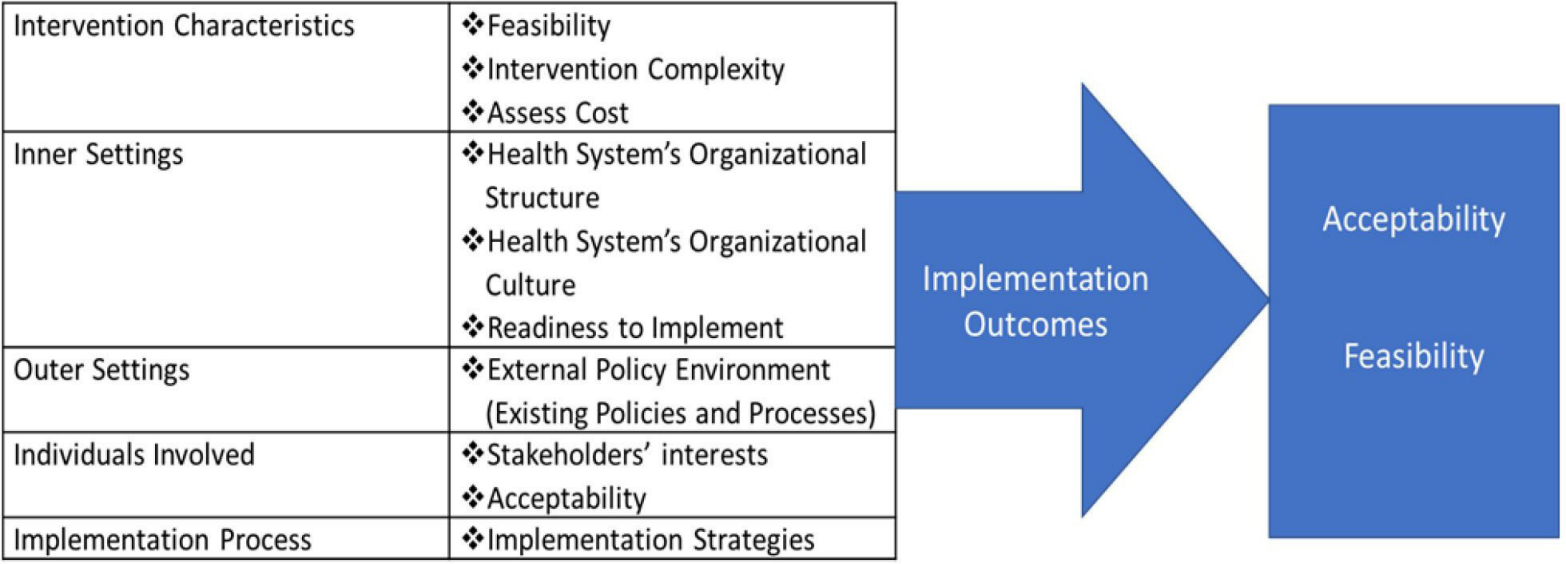
Implementation evaluation guided by the Consolidated Framework for Implementation Research.

The Consolidated Framework for Implementation Research (CFIR)^22^ and RE-AIM framework^23^ will guide our implementation research approach. The CFIR is a determinant framework made of five domains and multiple constructs. These domains are intervention characteristics, outer setting, inner setting, individuals’ characteristics and implementation process. Specifically related to our study, we identified some constructs of each domain that will facilitate the understanding of implementing FCM in our setting (figure 4). The CFIR will be used to guide and structure our implementation research approach.

### Data collection methods

To evaluate the outcome measures, qualitative and quantitative data will be collected. We will qualitatively evaluate the acceptability and feasibility of FCM using semistructured interview guides that are developed by CFIR. Specifically, we will conduct focus group discussions (FGDs) and key informant interviews (KIIs) among stakeholders of interest in each state prior to and after the trial’s commencement. Following the trial, stakeholders will be informed of the preliminary findings prior to the FGDs and KIIs. The stakeholders are general postpartum women with and without anaemia, postpartum women with past or current anaemia treatment, partners of postpartum women, healthcare personnel (HCPs) (eg, doctors, nurses, pharmacists, laboratory technologists, community health officers etc), health facility managers, community leaders and policymakers. The aim of the baseline assessment is to prospectively identify contextual barriers and facilitators to the trial implementation, while the endline will evaluate barriers and facilitators towards post-trial adoption, scale-up and sustainability. The sampling of respondents will be purposive but cut across various categories of stakeholders in the three levels of the health system in the various states.

FGDs were chosen as they enable members within stakeholder groups to interact, resulting in a comprehensive description of stakeholders’ perceptions of implementation outcomes.^23^ KIIs provide an opportunity for in-depth one-on-one discussions and are more practical to arrange for the identified stakeholders.^24^ We aim to conduct 48 KIIs and 12 FGDs, or until data saturation has been reached (a point in data collection when no additional issues or insights are identified from interviewing additional participants). Trained independent research assistants will conduct all interviews using purpose-designed interview guides specifically for each group of stakeholders. Verbal consent will be obtained from each participant before the start of every interview. Audio recordings will be made only with permission and transcribed verbatim.

Acceptability and feasibility will be assessed quantitatively using the Acceptability of Intervention Measure (AIM) and Feasibility of Intervention Measure (FIM) tools.^25^ These self-administered electronic survey tools will be completed through REDCap by HCPs administering FCM to postpartum women across all states, both before and after the trial.

Triangulation of the quantitative and qualitative findings will be conducted to provide a comprehensive understanding of the implementation outcomes. The quantitative data from the AIM and FIM tools will provide numerical measures of acceptability and feasibility, while the qualitative data from the FGDs and KIIs will offer in-depth insights into the contextual factors influencing these outcomes. By integrating the findings from both data sources, we can identify areas of convergence and divergence, enhancing the validity and reliability of the results. This triangulation approach will allow us to develop a more nuanced understanding of the barriers and facilitators to implementing FCM in the study setting, informing future adoption, scale-up and sustainability efforts.

### Patient and public involvement

Before commencement of the trial, FGDs and KIIs were held at various health facilities and communities to discuss common maternal health issues identified by the women. Their opinions and perception of the proposed trial were also discussed. Based on the feedback, the MIB and fatigue scale were included as secondary trial outcomes.

### Implementation outcomes analyses

Thematic analysis will be undertaken. All transcribed interviews and focus groups will be thoroughly reviewed for accuracy and completeness. Subsequently, the dataset will be familiarised to identify patterns, differences and similarities. Two researchers with qualitative training will independently code a subset of the transcript deductively using CFIR^22^ and inductively to generate additional themes. Based on these codes, a codebook agreed on by both coders will be created and applied to the rest of the dataset. In an iterative process, themes and subthemes will be developed, reviewed and refined to accurately reflect the dataset’s meaning. Survey responses from AIM and FIM will be analysed according to Weiner *et al* guidelines^25^ using descriptive statistics as well as tests of difference regression.

## Ethics and dissemination

Ethical approval has been obtained from the National Health Research Ethics Committee, Nigeria, (NHREC/01/01/2007-07/09/2022), teaching hospitals and the States’ health ethics boards. Approval was also obtained from the National Agency for Food and Drug Administration and Control. The trial is duly registered in the International Standard Randomised Controlled Trial Number (ISRCTN51426226) registry.

Written consent for participation including consent for community tracking will be obtained from all eligible women. Women aged between 15 and 18 years of age will be considered able to provide consent as they are considered emancipated for the purpose of this study according to guidelines of the NHREC and the 2016 WHO International Ethical Guidelines for Health-related Research Involving Humans. Additional consent for de-identified data and samples will be obtained for future studies or data sharing with secondary researchers. Confidentiality of data obtained from participants will be maintained throughout the trial. All the data collected at the study sites on REDCap will be stored centrally in a passworded electronic database by the senior data manager who will be the only one with access to the blinded data of all participants collated centrally. Any approved modification to the protocol will be communicated to the investigators and retraining of study staff on the amended protocol will be done as necessary.

Findings of this research will be shared by presentation at various scientific conferences in the fields of obstetrics, haematology and public health, publication in peer-reviewed journals, and engagement with healthcare providers and relevant policymakers globally. Community engagement events and press releases will be used to communicate results with the public and study participants.

### Trial status

The current protocol is V 2.0, 18 January 2024. The first participant was enrolled on 7 December 2022 and recruitment is projected to end by June 2024. The trial registration dataset is available on: https://www.isrctn.com/ISRCTN51426226.

### Ancillary and post-trial care

All participants are covered by clinical trial insurance in case of severe adverse drug events arising from the use of trial drugs. There are no specific plans for post-trial care.

## Data Availability

Data sharing not applicable as no datasets generated and/or analysed for this study.
