# Supplementary material for: Intravenous ferric carboxymaltose versus oral ferrous sulphate for the treatment of moderate to severe postpartum anaemia in Nigerian women (IVON-PP): protocol for an open-label randomised controlled type 1 hybrid effectiveness-implementation trial
Source: BMJ Open. 2024 Aug 14;14(8):e086553. doi: 10.1136/bmjopen-2024-086553 (PMC11331826; doi:10.1136/bmjopen-2024-086553)
Supplement: Supplementary file 1 [file bmjopen-2024-086553.reviewer_comments.pdf]

PEER REVIEW HISTORY

BMJ Open publishes all reviews undertaken for accepted manuscripts. Reviewers are asked to complete a checklist review form and are provided with free text boxes to elaborate on their assessment. These free text comments are reproduced below.

ARTICLE DETAILS

**TITLE (PROVISIONAL)**	Intravenous ferric carboxymaltose versus oral ferrous sulphate for the treatment of moderate to severe postpartum anemia in Nigerian women (IVON-PP): protocol for an open-label randomized controlled type I hybrid effectiveness-implementation trial.
**AUTHORS**	Afolabi, Bosede; Adaramoye, Victoria; Adeyemo, Titilope; Balogun, M; Mitchell, Eleanor; Walker, Kate; Akinajo, Opeyemi Rebecca; Abioye, Ibraheem; Banke-Thomas, Aduragbemi; Babah, Ochuwa; Chieme, Chisom Florence; Oshodi, Yewande; Quao, Rachel; Eboreime, Ejemai; Ogunsola, Folasade

VERSION 1 - REVIEW

**REVIEWER NAME**	Tuuli, Methodius G.
**REVIEWER AFFILIATION**	
**REVIEWER CONFLICT OF INTEREST**	None.
**DATE REVIEW RETURNED**	14-Apr-2024

**GENERAL COMMENTS**	This is an important study given the high prevalence of iron deficiency anemia in the postpartum period particularly in low- and middle-income countries. A few comments:<br><br>1. Provide a rationale for why inclusion is only based on hemoglobin level? Although iron deficiency is the most common etiology of anemia during pregnancy and postpartum it is important to confirm with a ferritin level prior to intravenous treatment.<br>2. Provide a justification for why the oral iron is dosed twice daily, when studies suggest that every other day dosing may have equivalent efficacy and less side effects?<br>3. Assessment of the implementation aspects is critical to scaling up if the trial results are supportive of intravenous iron.

**REVIEWER NAME**	Hamm, Rebecca
**REVIEWER AFFILIATION**	University of Pennsylvania, department of obstetrics and gynecology
**REVIEWER CONFLICT OF INTEREST**	None.
**DATE REVIEW RETURNED**	01-May-2024

**GENERAL COMMENTS**	This manuscript describes a research protocol for a type I hybrid effectiveness-implementation RCT. I am looking forward to seeing its results. Some recommendations to improve the manuscript prior to publication:<br><br>1. The title makes it seem as if this paper will present the results. Make it clear in the title that this is the study protocol.

	2. Can the title also call the study by its more rigorous design name - a type I hybrid effectiveness implementation RCT?<br>3. Consider use of the word “concurrent” to replace “alongside” throughout.<br>4. Timing and location of IVFe administration should be clarified.<br>5. Retention strategies - the specification of “male partners” is striking. Consider removing the word male.<br>6. Timing of performance of the implementation measures should be further described. When are these focus groups taking place? Only with patients in the IV iron arm or both? When are the surveys being distributed during the patients care? To all patients or only a subset? Only once or as a repeated measure?<br>7. Have the authors considered allowing their qual and quan implementation measures to interact and mix to enhance the rigor of their findings?<br>8. Can the authors describe what they plan to do with the implementation findings in relationship to the other aims of the work?

VERSION 1 – AUTHOR RESPONSE

Reviewer: 1

1. Provide a rationale for why inclusion is only based on hemoglobin level? Although iron deficiency is the most common etiology of anemia during pregnancy and postpartum it is important to confirm with a ferritin level prior to intravenous treatment.

Response.

Thank you for the comment. We are aware of assessing ferritin level and other markers prior to intravenous iron treatment in addition to blood haemoglobin level to determine iron deficiency anemia in adherence to the WHO recommendation (World Health Organization. Guideline on haemoglobin cutoffs to define anemia in individuals and populations. Geneva: World Health Organization; 2024). In the trial, we collect samples to check ferritin, other markers and the hemoglobin concentration at enrolment. However, due to the relatively long turnaround time of results, which could be as long as two weeks in our setting, the results are not available till weeks after enrolment. We use baseline hemoglobin levels to screen postpartum women, being a simple, inexpensive and an easy biomarker to measure in a clinical trial with a large sample size. Iron deficiency is considered the most common cause of anemia in pregnancy and in the postpartum, the probability of a woman being iron deficiency is rather high to warrant treating for iron deficiency before the iron deficiency may be confirmed (World Health Organisation. Anaemia 2023 [Available from: https://www.who.int/news-room/fact-sheets/detail/anemia). Also, the high risk of loss to follow up among postpartum women is considered and thus treatment is administered, and modalities instituted to ensure she is followed up throughout the specified time in the trial (Wilcox A, Levi EE, Garrett JM. Predictors of non-attendance to the postpartum follow-up visit. Maternal and Child Health Journal. 2016 Nov; 20:22-7).

2. Provide a justification for why the oral iron is dosed twice daily, when studies suggest that every other day dosing may have equivalent efficacy and less side effects?

Response.

The clinical trial is on treatment of women with moderate to severe anemia. Based on the in-country guideline for management of anemia, for treatment it is to be administered as a twice daily dosing and the once daily dosage is for prophylaxis. The trial is evaluating safety of the drug dosages and will give further knowledge on this, when it is completed (Afolabi B, Ogunbode O, Ezechi O, Ogu R, Agboghoroma C, Aboyeji A, et al. Management and the Prevention of Anemia in Pregnancy: SOGON Clinical Practice Guideline. Tropical Journal of Obstetrics and Gynaecology. 2023;40(2):64-9).

Reviewer: 2

1. The title makes it seem as if this paper will present the results. Make it clear in the title that this is the study protocol.

2. Can the title also call the study by its more rigorous design name - a type I hybrid effectiveness implementation RCT?

Response.

The title was, Intravenous ferric carboxymaltose versus oral ferrous sulphate for the treatment of moderate to severe postpartum anemia in Nigerian women (IVON-PP): an open label randomized controlled trial alongside an implementation study. It now reads, “Intravenous ferric carboxymaltose versus oral ferrous sulphate for the treatment of moderate to severe postpartum anemia in Nigerian women (IVON-PP): protocol for an open-label randomized controlled type I hybrid effectiveness-implementation trial”. Please see Page 1, lines 1-3.

3. Consider use of the word “concurrent” to replace “alongside” throughout.

Should this be changed based on the suggestion?

Response.

Thank you for this suggestion,

1. Intravenous ferric carboxymaltose versus oral ferrous sulphate for the treatment of moderate to severe postpartum anemia in Nigerian women (IVON-PP): an open label randomized controlled trial alongside an implementation study. This now reads, “Intravenous ferric carboxymaltose versus oral ferrous sulphate for the treatment of moderate to severe postpartum anemia in Nigerian women (IVON-PP): protocol for an open-label randomized controlled type I hybrid effectiveness-implementation trial”. Please see Page 1, Lines 1-3.

2. To evaluate the acceptability and feasibility of using intravenous FCM in treating postpartum anemia in Nigeria by conducting an alongside implementation study. This now reads, to evaluate the acceptability and feasibility of using intravenous FCM in treating postpartum anemia in Nigeria by conducting a concurrent implementation study. Please see Page 4, Lines 22-23.

4. Timing and location of IVFe administration should be clarified.

Response.

Thank you for your comments on the timing and location of intravenous iron administration. It was initially stated as “the intervention is FCM given as a single dose of 20mg/kg up to a maximum of 1000mg diluted in 200 ml Normal Saline and infused over a minimum of 15-20 minutes”. For clarity it now reads, “Intravenous iron is administered within the time allotted for enrolment, that is within 6-48hour after delivery. Eligible women randomized to the intervention arm (FCM) are administered intravenous iron at the dedicated ward or daycare room, where she is also observed after the drug administration”. Please see Page 6, Line 18–21.

5. Retention strategies - the specification of “male partners” is striking. Consider removing the word male.

Response.

Thank you for your comments,” to minimize loss to follow up, counselling sessions are extended to male partners and family members when necessary, during follow-up visits”. It now reads,” to minimize loss to follow up, counselling sessions are extended to partners and family members when necessary, during follow-up visits”. Please see Page 11, Line 9.

6. Timing of performance of the implementation measures should be further described. When are these focus groups taking place? Only with patients in the IV iron arm or both? When are the surveys being distributed during the patients care? To all patients or only a subset? Only once or as a repeated measure?

Response.

We have clarified that these measures will be taken before and after the trial from various stakeholder groups. We listed the stakeholders involved including patients, healthcare workers, policymakers, community representatives etc. Please see Page 14, Lines 21-26; and Page 15, lines 1-8

7. Have the authors considered allowing their qual and quan implementation measures to interact and mix to enhance the rigor of their findings?

Response.

We have described our triangulation approach on Page 15, line 23 to page 16, line 5.

8. Can the authors describe what they plan to do with the implementation findings in relationship to the other aims of the work?

Response.

Page 16 lines 1-5 now reads, " By integrating the findings from both data sources, we can identify areas of convergence and divergence, enhancing the validity and reliability of the results. This triangulation approach will allow us to develop a more nuanced understanding of the barriers and facilitators to implementing FCM in the study setting, informing future adoption, scale-up, and sustainability efforts."
